# The effect of fumaric acid on ruminant enteric methane emission and ruminal volatile fatty acids concentration: a meta-analysis

**DOI:** 10.1093/jas/skaf362

**Published:** 2025-10-22

**Authors:** Muhammad I Malik, Maria T Capucchio, Bereket Z Tunkala, M Muneeb, Chris M Ncho, Lizhuang Hao, Long Cheng

**Affiliations:** Department of Veterinary Sciences, University of Torino, Torino 10095, Italy; Department of Veterinary Sciences, University of Torino, Torino 10095, Italy; School of Agriculture, Food, and Ecosystem Sciences, Faculty of Science, The University of Melbourne, Dookie College, Victoria 3647, Australia; Department of Animal Nutrition, Faculty of Animal Production and Technology, University of Veterinary and Animal Sciences, Lahore 54000, Pakistan; Animal Nutrition, Department of Environmental Systems Science, Institute of Agricultural Sciences, ETH Zürich, Zürich 8092, Switzerland; Key Laboratory of Plateau Grazing Animal Nutrition and Feed Science of Qinghai Province, Qinghai University, Xining 810016, China; School of Agriculture, Food, and Ecosystem Sciences, Faculty of Science, The University of Melbourne, Dookie College, Victoria 3647, Australia

**Keywords:** beef, cattle, fumarate, goat, greenhouse gas, sheep

## Abstract

Methane emitted by ruminants represents an energy loss from feed intake and contributes to global warming. Fumaric acid (FA), a key intermediate in rumen metabolism, acts as an alternative electron acceptor and offers a potential strategy to reduce methane production. This meta-analysis systematically evaluated the effects of FA supplementation on enteric methane emissions and rumen fermentation in ruminants. Thirteen peer-reviewed studies met the inclusion criteria, contributing 22 effect sizes from 13 studies: six on cattle (dairy and beef cattle), seven on small ruminants (sheep and goats). Effect sizes were calculated as mean difference (MD) for methane yield (g/kg dry matter intake [DMI]), relative mean difference (RMD) for methane production (g/day) and DMI (kg/day), and standardized mean difference (SMD) for volatile fatty acids. A multilevel meta-analysis model was used to account for study-level variation. FA supplementation had no effect on DMI (*P *= 0.25; RMD = –2.75) but significantly reduced methane production (*P *= 0.005; RMD = –19.21). Meta-regression showed that increase in FA (g/kg DMI) decreases in methane production by 0.272% (*P *= 0.02). Methane production was significant in small ruminants (*P *= 0.002; RMD = –24.67) but not in cattle (*P *= 0.52; RMD = –7.03). The effectiveness of FA in reducing methane production was not (*P *> 0.05) affected by variations in dietary forage, concentrate, or neutral detergent fiber (NDF) content in FA-supplemented animals. Methane yield decreased (*P *= 0.001; MD = –1.954) in FA-supplemented animals, while the efficacy of FA was not influenced (*P *> 0.05) by diet composition (forage %, concentrate %, NDF %). FA had no effect on ruminal acetate (*P *= 0.49; SMD = –0.299), but increased propionate (*P *= 0.01; SMD = 0.970). In summary, FA supplementation did not affect DMI but reduced methane production and yield in small ruminants. While in cattle, FA supplementation may have limited impact on methane emission.

## Teaser

Fumaric acid supplementation reduces methane emissions in small ruminants (sheep and goat); however, FA supplementation is less effective in reducing methane emissions in cattle (beef and dairy).

## Introduction

The agriculture sector contributes approximately 10–12% of global anthropogenic greenhouse gas (GHG) emission (Smith et al., 2014). Due to unique digestive physiology, ruminants produce methane as a byproduct of enteric fermentation, contributing to approximately 40% of the agricultural sector’s carbon footprint and around 6% of total global GHG emissions. Therefore, developing nutritional or management approaches to help mitigate methane emissions from ruminant livestock is important (Smith et al., 2022).

Implementing methane mitigating strategies and monitoring systems in farm animals can provide both long-term environmental benefits and short-term economic gains (McGinn et al., 2004; Cheng, 2020). Over the past decade, several strategies have been identified, each with varying level of effectiveness in reducing methane emission from livestock. Recent advancements in understanding the metabolic pathways of methanogenesis in rumen present new opportunities to target the methanogenesis through multiple approaches (Honan et al., 2021).

Redirecting hydrogen use away from methane production toward other metabolic pathways is a promising strategy for methane reduction (Guyader et al., 2017). Methanogenesis serves as a primary mechanism for disposal of hydrogen in rumen fermentation. However, shifting rumen volatile fatty acid (VFA) concentration from acetate to propionate also acts as a hydrogen sink, potentially reducing methane emissions (Benchaar et al., 2001).

Organic acids such as malate and fumaric acid (FA), which are intermediates of the citric acid cycle, naturally occur in plants at concentrations of 20–80 g/kg of dry matter (Jones and Barnes, 1967). A study evaluated 15 propionate precursors and found that FA and acrylate consistently reduced methane production in batch cultures with FA proving to be more effective than acrylate (Newbold et al., 2005). Moreover, a consistent reduction in methane emissions was reported when using FA in artificial rumen models (Ungerfeld et al., 2007). Certain bacteria also utilize FA to produce propionate via a reverse citric acid cycle (Castillo et al., 2004). Fumarate reduction (i.e., gain of electrons or hydrogen) involves the use of hydrogen molecules, creating competition of hydrogen either to fuel methanogenesis or to redirect hydrogen toward propionate production. This competition ultimately decreases the availability of hydrogen for methanogenesis in the rumen, consequently reducing methane emissions (Asanuma et al., 1999).

The literature reports conflicting results regarding the impact of FA on ruminal propionate concentration, with some studies observing no contribution to ruminal propionate concentration (De Nardi et al., 2014; Vyas et al., 2015). In vivo data indicated that FA did not influence enteric methane emission in beef cattle (McGinn et al., 2004; Beauchemin and McGinn, 2006), but it reduced methane emission in steers (Bayaru et al., 2001), sheep (Newbold et al., 2002), and goats (Li et al., 2018; Li et al., 2021). Due to these inconsistent findings and lack of any meta-analysis published on FA supplementation and its effects on enteric methane emissions in ruminants, a meta-analysis was performed with objectives of evaluating the effects of FA supplementation on enteric methane emissions and VFA concentration in the rumen, as well as quantifying its potential for methane mitigation.

## Materials and Methods

### Search strategy

A systematic literature search was conducted on 18th August 2024 to retrieve the relevant research titles. For literature search, two databases were selected 1) PUBMED (https://www.ncbi.nlm.nih.gov/pubmed/) and 2) SCOPUS (https://www.scopus.com/home.uri). The search terms for both databases were [(fumarate OR fumaric acid) AND (methane OR CH4)].

### Inclusion and exclusion criteria

Studies were considered eligible if they reported enteric methane emissions for both control and treatment groups. Studies were excluded if they were involved in vitro FA supplementation or if either group received additional methane-mitigating agents. Only peer-reviewed articles published in English were included. A total of 133 titles were identified through PubMed and 261 through Scopus, with one additional study located via a reference list review. Following the initial retrieval, manual screening was conducted, and 110 duplicate records were removed. Irrelevant articles, including in vitro studies (*n* = 54), pig-related studies (*n* = 3), reviews, and theses (*n* = 17), were also excluded. Subsequently, abstracts of the remaining articles were carefully reviewed, and further ineligible studies were eliminated based on predefined criteria. Any discrepancies during the selection process were resolved through discussion among the authors. A detailed overview of the inclusion and exclusion process is illustrated in the PRISMA flow diagram (Supplementary Figure S1, see online supplementary material for a color version of this figure). One manuscript in press was identified during the internal revision, which met the inclusion criteria and was included in the analysis; the data were then reanalyzed (Dong et al., 2025), a total of 13 studies, comprising 22 effect sizes, met the inclusion criteria. Among these, three studies focused on dairy cattle, three on beef cattle, three on lambs, and four on goats. Methane quantification was conducted using a respiratory chamber in 11 studies, SF6 in one study, and tunnel ventilation in one study. The experimental designs included Latin square design in seven studies, randomized block design in four studies, and completely randomized design in two studies. All studies were conducted under intensive feeding systems, except for one study that involved grazing cows (Supplementary Table S1).

### Data collection

The raw data for the means of control and treated groups for DMI, methane production, methane yield, and VFA were collected directly from the results reported in each eligible study. Additionally, information on the number of animals and measures of variability, including standard deviation (SD), standard error of mean, or standard error of difference, was also extracted from the corresponding tables. Study characteristics such as FA dose, animal type, design of experiment, types of FA supplementation, forage %, concentrate % of the diet, and neutral detergent fiber % (NDF) of the diet were also recorded from the reviewed articles. However, this meta-analysis did not evaluate methane intensity (e.g., methane per unit of milk or weight gain), as most included studies did not report consistent production performance metrics such as milk yield or average daily gain. Therefore, methane yield and production were used as proxies for mitigation efficacy.

The DMI values reported by McGinn et al. (2004) and Beauchemin and McGinn (2006) included two types: DMI for the entire experimental period and DMI measured during the time animals were in the respiratory chamber. In the current meta-analysis, we used the DMI for the complete trial, as one of the primary objectives was to assess the long-term effects of FA on DMI. The FA dose was standardized to a common unit of g/kg DMI, using DMI data from the full experiment to maintain consistency across all conversions. All studies reported DMI, except Newbold et al. (2002), which presented organic matter intake instead; therefore, organic matter intake was included in our database.

In Bayaru et al. (2001) rumen VFA data were presented at 0, 2, and 5 h after feeding. For consistency, we extracted values reported at 5 h post-feeding. Methane production was generally reported in g/day, and methane yield in g/kg DMI. While a few reported values in liter/day. These were converted to g/day using the molecular weight and volume of methane, assuming that one mole of methane (16.0 g) occupies 22.4 liters (Appuhamy et al., 2013). The variance in Kolver and Aspin (2006) and Wood et al. (2009) presented as standard error of mean difference; for these studies, SD values were calculated using the RevMan calculator (Version 5.4, The Cochrane Collaboration, 2020).

### Data analysis

Three types of effect size were calculated to reflect the differences in response variables. Mean difference (MD), defined as MD = treatment mean − control mean, was used for methane yield (g/kg DMI). For methane production (g/day) and DMI (kg/day), relative mean difference (RMD) was applied.


$$\text{RMD}=\frac{\text{Treatment mean}-\text{Control mean}}{\text{Control mean}}\times 100$$

The RMD, a dimensionless variable, was used to account for large variations arising from multi-species data. Additionally, it enables readers to interpret the results in terms of percentage changes in methane production (Malik et al., 2024). The standardized mean difference (SMD) was used for VFA. It was calculated as follows:


$$\mathrm{SMD}=\frac{\mathrm{Tretament}\;\mathrm{mean}-\mathrm{Control}\;\mathrm{mean}}{\mathrm{SD}}$$

A positive SMD indicates that the treatment group had a higher mean than the control group, while a negative SMD suggests that the control group had a higher mean than the treatment group (Andrade, 2020). The SMD is a statistical technique commonly used in meta-analysis to compare and combine results from different studies that employ varying measurement scales (Higgins et al., 2022). Effect size was considered statistically significant at *P* < 0.05 and were regarded as a tendency when 0.05 ≤ *P* < 0.10. A multilevel meta-analysis model was fitted to the data. The Q-test for heterogeneity (Cochran, 1954) and the *I*^2^ statistic (Higgins and Thompson, 2002) were calculated as follows:


$$I^{2}=\frac{Q-(k-1)}{Q}\times 100$$

where *Q* is the Chi^2^ statistic, and *k* is the number of studies included in the meta-analysis.

A multilevel meta-analysis approach was used to account for the hierarchical structure of the data, where effect sizes were nested within studies. This was necessary because some studies reported more than one effect size. The method allows for the partitioning of variance within and between studies, enabling more accurate estimation of treatment effects. In this framework, effect sizes extracted from the same study were treated as nested and considered higher-level units. By accounting for variation at multiple levels, the multilevel meta-analysis approach improves the precision of effect size estimates and helps identify sources of heterogeneity (Cheung, 2014; Assink and Wibbelink, 2016).

The model was estimated using restricted maximum likelihood (REML), with effect sizes as the dependent variable and study characteristics as moderators. Meta-regression was conducted to evaluate potential effect size influencers.

The model of our multilevel meta regression was as described by Harrer et al. (2021). Precisely, the equation of the model was the following


$${\hat{\theta }}_{ij}=\theta +\;{\beta x}_{i}+\;\zeta_{(2)ij}+\zeta_{(3)j}+\;\epsilon_{ij}$$

where ${\hat{\theta }}_{ij}\;$ is an estimate of the *i*th effect size nested within the *j*th study; θ is the overall mean true effect size; ${\beta x}_{i}\;$ is the effect of the predictor variable $x_{i}$ (FA dose, NDF, forage, concentrate) with β as its regression coefficient; $\zeta_{(2)ij}\;$ is the heterogeneity associated with the *i*th effect size nested within the *j*th study; $\zeta_{(3)j}\;$ is the heterogeneity associated with the *j*th study; $\epsilon_{ij}\;$ is the sampling error of the *i*th effect size from the *j*th study.

To avoid multicollinearity, individual moderators were added to the model in a stepwise manner. In the initial analysis, animal type (sheep, goat, dairy cattle, and beef cattle) was included as a moderator. No major differences were observed between small ruminants (sheep and goat) and cattle (dairy and beef). Due to the limited number of studies on enteric methane emissions, two groups of small ruminants (goat, sheep) and cattle (beef and dairy cattle) were created and used in the final analysis. This grouping enhances the generalizability of the findings and increases the number of studies per group, thereby improving the statistical power and reliability of the meta-analysis.

To assess potential publication bias, funnel plot asymmetry was evaluated using the Egger’s regression test (Sterne and Egger, 2005), with the standard error of the observed outcomes as the predictor. All analyses were conducted in R (version 4.4.0) (R Core, 2020) using the metafor package (version 4.6.0) (Viechtbauer, 2010). Orchard plots were generated using the orchaRd package (version 2.0) (Nakagawa et al., 2023).

## Results

The descriptive characteristics of the dataset are presented in Supplementary Table S2. The average dose of FA (g/kg DMI) was 17.86 for beef cattle, 27.92 for dairy cattle, 65.31 for lambs, and 19.04 for goats. The concentrate percentage of the diet was 83.33% for beef cattle, 69.50% for dairy cattle, 95.0% for lambs, and 50.38% for goats. The ether extract content of the diet ranged from 2.10 to 4.10% on a DM basis. The CP content ranged from 11.9 to 17.6%, while the NDF content ranged from 35.4 to 40.8% of the diet.

A total of 19 effect sizes from 12 studies were included in the meta-analysis for DMI (Supplementary Figure S2A, see online supplementary material for a color version of this figure). FA had no effect on DMI (*P* = 0.25, RMD = −2.75) (Table 1). The heterogeneity (*I*^2^) was 0%, and the Q statistic was also non-significant (*P* = 0.65, Q = 7.743). Funnel plot asymmetry was non-significant (Supplementary Figure S3A, see online supplementary material for a color version of this figure), and Egger’s regression test also indicated no asymmetry (*P* = 0.66). Meta-regression results showed that FA dose (g/kg DMI) also has no effect (*P* = 0.07, RMD = −0.123) on DMI. FA had no effect across animal types (*P* > 0.05), with cattle (*P* = 0.50, RMD = −2.71) and small ruminants (*P* = 0.38, RMD = −2.72) showing similar results. However, an increased proportion of forage in the diet reduced DMI in animals supplemented with FA (*P* = 0.01, RMD = −0.308). Similarly, the proportion of concentrate increased DMI in FA-supplemented animals (*P* = 0.01, RMD = 0.308).

**Table 1.** The summary statistics for fumaric acid supplementation and its effects on dry matter intake

Parameters	Effect size	Intercept	SE	*T* value	*N*	*P*-value	95% CI	*I* ^2^
DMI	−2.752	–	2.355	−1.168	19	0.257	−7.701 to 2.196	0.0
Fumaric dose	−0.123	1.790	0.066	−1.864	19	0.079	−0.263 to −0.016	–
*Types of animals*								
Cattle	−2.716	–	3.975	−0.683	6	0.503	−11.104 to 5.670	–
Small ruminants	−2.743	–	3.091	−0.887	14	0.387	−9.266 to 3.778	–
*Diet*								
Forage %	−0.308	16.205	0.103	−2.978	14	0.011	−0.534 to −0.082	–
Concentrate %	0.308	−14.630	0.103	2.978	14	0.011	0.082 to 0.534	–
NDF %	−0.743	23.827	0.713	−1.041	14	0.318	−2.298 to 0.811	–

SE = standard error, *N* = number of effect size, 95% CI = 95% confidence interval; all effect sizes are reported as RMD, calculated as (MD/control mean) × 100, as described in the Methods section.

For methane production, 22 effect sizes from 13 studies met the inclusion criteria. Methane production was reduced (*P *= 0.005, RMD = −19.21) (Table 2, Supplementary Figure S2B, see online supplementary material for a color version of this figure). Heterogeneity was high, with *I*^2^ = 99.84%, and the Q statistic was significant (*P *< 0.001). Funnel plot asymmetry was not significant (Supplementary Figure S3B, see online supplementary material for a color version of this figure), and Egger’s regression test also showed no evidence of asymmetry (*P *= 0.12). The meta-regression model indicated that the dose of FA supplementation decreased methane production (*P *< 0.001). For each 1 g/kg DMI increase in FA, methane production declined by 0.272%. Among animal types, methane production was reduced in small ruminants (*P *= 0.002, RMD = −24.67), whereas no reduction was observed in cattle (*P *= 0.52, RMD = −7.039). The forage, concentrate and NDF contents of the diet had no influence (*P *> 0.05) on efficacy of FA in methane production in small ruminants and cattle.

**Table 2.** The summary statistics for fumaric acid supplementation and its effects on methane production

Parameters	Effect size		Intercept	SE	*T* value	*N*	*P*-value	95% CI	*I* ^2^
Methane production	−19.215		–	6.187	−3.105	22	0.005	−32.083 to −6.348	99.84
Fumaric dose	−0.272		−10.045	0.109	−2.490	22	0.021	0.500 to −0.044	–
*Types of animals*									
Cattle	−7.039		–	10.986	−0.640	7	0.528	−29.956 to 15.876	–
Small ruminants	−24.678		–	7.268	−3.395	15	0.002	−39.840 to −9.515	–
*Diet*									
Forage %	0.040		−20.011	0.205	0.198	17	0.845	−0.396 to 0.477	–
Concentrate %	−0.040		−15.938	0.251	−0.198	17	0.845	−0.477 to 0.396	–
NDF %	−0.837		14.827	1.134	−0.738	16	0.472	−3.270 to 1.595	–

SE = standard error, *N* = number of effect size, 95% CI = 95% confidence interval; all effect sizes are reported as RMD, calculated as (MD/control mean) × 100, as described in the Methods section.

A total of 18 effect sizes from 11 studies were included in the meta-analysis for methane yield (g/kg DMI). FA supplementation reduced methane yield (*P *= 0.001, MD = −1.95; Table 3, Supplementary Figure S2C, see online supplementary material for a color version of this figure). Heterogeneity was moderate, with *I*^2^ = 77.66%, and the Q statistic was significant (*P *< 0.001, *Q* = 58.65). Funnel plot asymmetry was significant, and Egger’s regression test also confirmed this (*P *= 0.03; Supplementary Figure S3C, see online supplementary material for a color version of this figure). Meta-regression results indicated that FA dose (g/kg DMI) reduced methane yield (*P *= 0.03, MD = −0.026). Among animal types, methane yield was reduced in small ruminants (*P *= 0.001, MD = −2.615), while no effect was observed in cattle (*P *= 0.15, MD = −1.150). Variation in forage, concentrate, or NDF content of the diet did not affect the efficacy of FA in reducing methane yield (*P* > 0.05).

**Table 3.** The summary statistics for fumaric acid supplementation and its effects on methane yield

Parameters	Effect size	Intercept	SE	*T* value	*N*	*P*-value	95% CI	*I* ^2^
Methane yield	−1.954	–	0.521	−3.744	18	0.001	−3.055 to −0.853	77.66
Fumaric dose	−0.026	−1.035	0.011	−2.326	18	0.035	−0.050 to 0.002	–
*Types of animals*								
Cattle	−1.150	–	0.766	−1.501	7	0.152	−2.774 to 0.473	–
Small ruminants	−2.615	–	0.681	−3.838	11	0.001	−4.059 to −1.171	–
*Diet*								
Forage %	−0.025	−3.529	0.029	0.886	15	0.391	−0.037 to 0.089	–
Concentrate %	−0.025	−0.938	0.029	0.886	15	0.391	−0.089 to 0.037	–
NDF %	0.187	−8.545	0.172	1.091	14	0.296	−0.187 to 0.562	–

SE = standard error, *N* = number of effect size, 95% CI = 95% confidence interval; all effect sizes are reported as MD, calculated as: MD = Treatment mean − Control mean.

A total of 15 effect sizes from 10 studies were included in the analysis. The observed SMD ranged from −3.29 to 5.20, with most estimates being negative (50%). The estimated average SMD based on the multilevel random-effects model was −0.299, indicating no statistically significant overall effect of FA on acetate concentration (*P *= 0.49; Table 4, Supplementary Figure S2D, see online supplementary material for a color version of this figure). The Q-test indicated a significant level of heterogeneity in the true outcomes (*Q* = 46.85, *I*^2^ = 76.35%). A funnel plot of the estimates is presented in Supplementary Figure S3D, see online supplementary material for a color version of this figure, and Egger’s test for asymmetry was also non-significant (*P *= 0.07). The meta-regression model showed that the dose of FA reduced acetate concentration (*P *= 0.01, SMD = −0.055). There were no differences in acetate concentration in both, cattle (*P *= 0.90, SMD = 0.087) and small ruminants (*P *= 0.36, SMD = −0.626) showing similar responses. Variations in dietary forage, concentrate, and NDF content neither enhanced nor attenuated (*P *> 0.05) the effect of FA on ruminal acetate concentration.

**Table 4.** The summary statistics for fumaric acid supplementation and its effects on rumen acetate concentration

Parameters	Effect size	Intercept	SE	*T* value	*N*	*P*-value	95% CI	*I* ^2^
Acetate	−0.299	–	0.428	−0.698	15	0.496	−1.218 to 0.619	76.35
Fumaric dose	−0.055	1.052	0.020	−2.624	15	0.010	−0.100 to −0.009	–
*Types of animals*								
Cattle	0.087	–	0.698	0.124	6	0.902	−1.421 to 1.595	–
Small ruminants	−0.626	–	0.659	−0.948	9	0.360	−0.205 to 0.799	–
*Diet*								
Forage %	0.034	−2.311	0.024	1.364	12	0.202	−0.021 to 0.089	–
Concentrate %	−0.034	1.087	0.024	−1.364	12	0.202	−0.089 to 0.021	–
NDF %	−0.090	−3.629	0.092	−0.973	11	0.355	−0.119 to 0.300	–

SE = standard error, *N* = number of effect size, 95% CI = 95% confidence interval; all effect sizes are reported as SMD, calculated as: SMD = (Treatment mean − Control mean)/SD.

A total of 15 effect sizes from 10 studies were included in the analysis. FA supplementation increased propionate concentration (*P *= 0.01, SMD = 0.970) (Table 5, Supplementary Figure S2E, see online supplementary material for a color version of this figure). Heterogeneity was moderate, with *I*^2^ = 63.69%, and the Q statistic was significant (*P *< 0.001, *Q* = 39.83). Egger’s regression test for funnel plot asymmetry (Supplementary Figure S3E, see online supplementary material for a color version of this figure) was non-significant (*P *= 0.15). Meta-regression results indicated that FA dose (g/kg DMI) has no influence on propionate (*P *= 0.06, SMD = 0.062). There were no differences in propionate concentration in cattle (*P *= 0.24, SMD = 0.492), while in small ruminants rumen propionate concentration was higher (*P *= 0.01, SMD = 1.363) FA treatment as compared to control. Dietary proportions of forage, concentrate, and NDF did not influence (*P *> 0.05) the impact of FA on ruminal propionate concentration.

**Table 5.** The summary statistics for fumaric acid supplementation and its effects on rumen propionate concentration

Parameters	Effect size	Intercept	SE	*T* value	*N*	*P*-value	95% CI	*I* ^2^
Propionate	0.970	–	0.344	2.818	15	0.013	0.231 to 1.709	63.69
Fumaric dose	0.062	−0.455	0.025	2.498	15	0.067	0.008 to 0.116	–
*Types of animals*								
Cattle	0.592	–	0.482	1.227	6	0.241	−0.449 to 1.633	–
Small ruminants	1.363	–	0.491	2.776	9	0.015	0.302 to 2.424	–
*Diet*								
Forage %	−0.009	1.401	0.017	−0.530	12	0.607	−0.047 to 0.029	–
Concentrate %	0.009	0.489	0.017	0.530	12	0.607	−0.029 to 0.047	–
NDF %	0.034	−0.486	0.087	0.392	11	0.703	0.163 to 0.232	–

SE = standard error, *N* = number of effect size, 95% CI = 95% confidence interval; all effect sizes are reported as SMD, calculated as: SMD = (Treatment mean − Control mean)/SD.

A total of 15 effect sizes from 10 studies were included in the rumen butyrate concentration. The estimated SMD based on the multilevel random-effects model did not differ significantly overall (Table 6, Supplementary Figure S2F, see online supplementary material for a color version of this figure), although it showed a decreasing trend (*P *= 0.07, SMD = −0.669). The Q-test indicated a significant level of heterogeneity in the true outcomes (*Q* = 40.25, *I*^2^ = 68.9%). A funnel plot of the estimates is shown in Supplementary Figure S3F, see online supplementary material for a color version of this figure, and Egger’s test was non-significant (*P *= 0.38). The meta-regression model indicated that an increase in FA dose reduced rumen butyrate concentration (*P *= 0.01, SMD = −0.058). Among animal types, there were no effects of FA on rumen butyrate concentration in cattle (*P *= 0.43, SMD = −0.428) while in in small ruminants a decreasing trend (*P *= 0.08, SMD = −0.908) was observed for rumen butyrate. Variations in dietary forage, concentrate, and NDF content neither enhanced nor attenuated (*P *> 0.05) the effect of FA on ruminal butyrate concentration.

**Table 6.** The summary statistics for fumaric acid supplementation and its effects on rumen butyrate concentration

Parameters	Effect size	Intercept	SE	*T* value	*N*	*P*-value	95% CI	*I* ^2^
Butyrate	−0.669	–	0.341	−1.960	15	0.070	−1.402 to 0.062	68.90
Fumaric dose	−0.058	0.740	0.020	−2.782	15	0.015	−0.103 to −0.013	–
*Types of animals*								
Cattle	−0.428	–	0.528	−0.811	6	0.432	−1.570 to 0.713	–
Small ruminants	−0.908	–	0.478	−1.987	9	0.080	−1.942 to −0.125	–
*Diet*								
Forage %	−0.012	0.480	0.014	−0.852	12	0.414	−0.045 to 0.020	–
Concentrate %	0.012	−0.787	0.014	0.852	12	0.414	−0.020 to 0.045	–
NDF %	−0.042	1.333	0.071	−0.600	11	0.562	−0.203 to 0.118	–

SE = standard error, *N* = number of effect size, 95% CI = 95% confidence interval; all effect sizes are reported as SMD, calculated as: SMD = (Treatment mean − Control mean)/SD.

## Discussion

Livestock farming contributes to GHG emissions, particularly enteric methane (Cheng, 2020). A range of methane mitigation strategies has been developed, with feed additives being among the most extensively researched and applied approaches (Hristov, 2024). Despite their potential, additives often present trade-offs, such as increased enteric hydrogen emissions, potential reductions in productivity, or inconsistent effects on net methane output. In particular, it is not uncommon for studies to report substantial reductions in absolute methane production (g/day), while methane yield (g/kg DMI), a more intake-adjusted and biologically meaningful measure, shows little or no change (Hristov, 2024). This divergence highlights the need to assess mitigation efficacy using both total emissions and feed efficiency-based metrics.

Biochemically, elevated ruminal hydrogen concentrations are a double-edged sword. While hydrogen accumulation reflects redirected fermentation pathways, it also represents an energetic loss to the host animal and serves as a critical substrate for methanogenesis. This shift toward a more reduced rumen redox environment may compromise normal fermentation dynamics and nutrient utilization (McAllister and Newbold, 2008). Among the various feed additives, organic acids, particularly FA, have received considerable attention due to their dual role as metabolic intermediates and hydrogen sinks. These compounds help redirect reducing equivalents toward propionate concentration, thereby lowering hydrogen availability for methanogenesis (McAllister and Newbold, 2008).

Newbold et al. (2005) systematically evaluated 15 propionate precursors and identified FA and acrylate as the most effective in reducing methane emissions in batch culture systems, with FA performing better than acrylate in artificial rumen conditions. Further in vitro evidence supports the consistency of FA in lowering methane output (Ungerfeld et al., 2007). However, in vivo results have been mixed. Several studies reported no significant effect of FA on enteric methane emissions in beef cattle (McGinn et al., 2004; Beauchemin and McGinn, 2006) and dairy cattle (Bayaru et al., 2001; Kolver and Aspin, 2006; Maigaard et al., 2024). In contrast, methane reductions have been observed in steers (Bayaru et al., 2001) and sheep (Newbold et al., 2002), suggesting that species-specific rumen physiology and dietary contexts may influence the effectiveness of FA as a methane mitigation agent.

Our meta-regression results indicated that increases in FA dose has no effects on DMI. These findings align with previous reports showing no effects of FA on DMI in beef cattle (McGinn et al., 2004). The FA supplementation significantly reduced methane production by 19.21% and methane yield by 1.95 g/kg DMI. Similar reductions in enteric methane have been observed in steers (Bayaru et al., 2001), sheep (Newbold et al., 2002), lambs (Wood et al., 2009), and goats (Yang et al., 2012; Li et al., 2018, 2021). These reductions are likely linked to two major biological mechanisms. First, FA contributes to methane mitigation through the succinate propionate pathway, where it is reduced to succinic acid and subsequently decarboxylated to propionate. This conversion consumes one mole of hydrogen per mole of FA, effectively acting as a hydrogen sink that reduces substrate availability for methanogenesis (Ungerfeld et al., 2007). Although studies have shown variable results regarding hydrogen redirection, many support the potential of FA to shift hydrogen utilization away from methane formation (Bayaru et al., 2001; Van Zijderveld et al., 2011). Our meta-analysis further supports this mechanism, showing a significant increase in propionate concentration (SMD = 0.970) and a positive dose–response relationship (SMD = 0.067) with FA supplementation. These results are consistent with findings from studies on steers (Bayaru et al., 2001), sheep (Newbold et al., 2002), beef cattle (Beauchemin and McGinn, 2006), and goats (Yang et al., 2012; Li et al., 2018, 2021). The second mechanism involves microbial modulation. The efficiency of FA conversion into propionate and the associated methane reduction may exceed the theoretical potential of hydrogen redirection alone. Yang et al. (2012) and Van Zijderveld et al. (2011) proposed that methane-suppressing effects of FA are not solely attributable to its role as a hydrogen sink. FA supplementation may also stimulate FA-utilizing bacterial populations, thereby enhancing the succinate–propionate pathway in the rumen (Zhou et al., 2012; Li and Guan, 2017). This microbial shift could increase the fermentation of other dietary substrates, such as starch and fiber, into propionate, while concurrently lowering the abundance of hydrogenotrophic methanogens (Yang et al., 2012).

The observed reduction in methane emissions (yield and production), alongside increased propionate concentration, suggests that propionogenesis can serve as an alternative hydrogen sink to methanogenesis, offering a more energy-efficient pathway for rumen fermentation (Ellis et al., 2008; Janssen, 2010; Guyader et al., 2017). Fumarate functions as an intermediate in the propionate synthesis pathway, facilitating propionate formation while competing with methanogens for available hydrogen (Yang et al., 2012). The methane-reducing potential of FA appears to be dose-dependent. Based on our raw data, average FA doses were 17.86, 27.92, 65.31, and 19.30 g/kg DMI for beef cattle, dairy cattle, sheep, and goats, respectively. Regression models indicated a significant reduction in methane production in small ruminants, whereas the non-significant effect observed in cattle may suggest that higher FA doses are required in cattle, possibly due to differences in diet composition or rumen environment, such as forage type and particle retention time.

Notable species-specific differences in effect sizes were also observed. In small ruminants, methane production was reduced by 24.67%, while in cattle the reduction was only 7.03%. This disparity may be attributed to physiological differences in rumen fermentation dynamics, microbial community structure, or feed digestion kinetics between small ruminants and cattle, which may influence the availability and utilization of hydrogen for competing pathways. However, it is important to acknowledge potential sources of bias. Some studies in our dataset, particularly those involving small ruminants, may have produced inflated effect sizes due to limited sample sizes or specific experimental conditions that do not fully reflect commercial dairy or beef production systems. These factors could have contributed to a larger overall estimated effect for FA supplementation across the meta-analysis. Such potential bias highlights the need for caution when generalizing findings across species.

To improve the reliability and applicability of future research, studies should aim to implement standardized experimental protocols and include sufficient replication across different ruminant types. This will help clarify species-specific responses to FA and enhance the accuracy of methane mitigation potential assessments under varying production systems. Increasing the proportions of forage, NDF, and concentrate in the diet has no influence on methane production or yield under the influence of FA supplementation, so it can be assumed that dietary factors neither enhanced nor attenuated the efficacy of FA on methane emission. The discrepancies between small ruminants and cattle could be associated to differences in rumen microbial populations, rates of fumarate metabolism, or the scale of hydrogen flow in the rumen compared to small ruminants. Additionally, adaptation of the rumen microbiota to fumarate metabolism may be less pronounced in cattle, limiting the efficacy of this intervention.

One of the major limitations of this meta-analysis is the limited number of studies available on FA supplementation as a methane-mitigating agent. Therefore, the results should be interpreted with caution. Another constraint was the inability to determine the exact amount of FA provided to the animal, due to a lack of detailed information in many of the included studies. Most reports did not specify the chemical form, purity, or actual intake of FA, which limited our ability to standardize across datasets. Future studies are required to consider detail reporting of FA composition and dosing to improve cross-study comparability and precision in estimating its effects.

## Conclusion

The findings of this meta-analysis suggest that FA supplementation has no significant effect on DMI in either small ruminants or cattle. However, FA supplementation resulted in a notable reduction in methane production and methane yield, by 19.21% and 1.95 g/kg DMI, respectively. Additionally, rumen propionate concentration increased, whereas acetate and butyrate concentrations were not significantly affected. Regression analyses suggest that supplementation of FA seems to be effective in reducing methane emissions in small ruminants. However, FA supplementation may be less effective in reducing methane emissions in cattle.

These species-specific differences may be attributed to physiological variation, or inadequate FA dosing in dairy and beef cattle studies. Although nutrient composition (forage, concentrate, and NDF) is known to influence methane emissions, our meta-analysis did not detect a significant effect. These findings should be interpreted in the context of the nutrient ranges represented in the included studies; effects may differ outside these ranges. Overall, the results support the potential of FA to contribute to methane mitigation in small ruminants, while highlighting the need for species-specific dosing strategies and more standardized research protocols in future studies.

## Supplementary Material

skaf362_Supplementary_Data

## Data Availability

All summarized data are presented in the tables, and the raw data will be made available upon reasonable request.

## Abbreviations:

DMI: dry matter intake
FA: fumaric acid
GHG: greenhouse gas
MD: mean difference
NDF: neutral detergent fiber
RMD: relative mean difference
SD: standard deviation
SMD: standardized mean difference
VFA: volatile fatty acids
