# Supplementary material for: The effect of fumaric acid on ruminant enteric methane emission and ruminal volatile fatty acids concentration: a meta-analysis
Source: J Anim Sci. 2025 Oct 22;103:skaf362. doi: 10.1093/jas/skaf362 (PMC12597141; doi:10.1093/jas/skaf362)
Supplement: skaf362_Supplementary_Data [file skaf362_supplementary_data.docx]

**Supplementary Table S1**

The database and studies characteristics for studies included in meta-analysis.

| Authors and Years | Fumaric acid g/kg DMI | Animal | DOE | Types of fumaric acid | CH_4_ quantification method | Forage % | Concentrate % | EE % | CP % | NDF % | Feeding system | Forage type |
| --- | --- | --- | --- | --- | --- | --- | --- | --- | --- | --- | --- | --- |
| Bayaru et al. (2001) | 20.0 | Beef cattle | CRD | FA | RC | 100 | 0 | 2.1 | 6.8 |  | Intensive | Sorghum silage |
| McGinn et al. (2004) | 9.2 | Beef cattle | LSD | FA | RC | 75 | 25 |  | 14.5 | 36.2 | Intensive | Barley silage |
| Beauchemin and McGinn (2006) | 24.4 | Beef cattle | LSD | FA | RC | 75 | 25 |  | 14.6 | 34.7 | Intensive | Barley silage |
| Kolver and Aspin (2006) | 48.4 | Dairy cattle | RCBD | Sodium fumarate | SF6 | 100 | 0 | 2.9 | 19.8 | 41.3 | Grazing | Ryegrass |
| Molano et al. (2008) | 40.0 | Lambs | RCBD | FA | SF6 | 95 | 5 | 2.2 | 17.6 | 40.8 | Intensive | Lucerne hay |
| Molano et al. (2008) | 60.0 | Lambs | RCBD | FA | SF6 | 95 | 5 | 2.2 | 17.6 | 40.8 | Intensive | Lucerne hay |
| Molano et al. (2008) | 80.0 | Lambs | RCBD | FA | SF6 | 95 | 5 | 2.2 | 17.6 | 40.8 | Intensive | Lucerne hay |
| Molano et al. (2008) | 100.0 | Lambs | RCBD | FA | SF6 | 95 | 5 | 2.2 | 17.6 | 40.8 | Intensive | Lucerne hay |
| Wood et al. (2009) | 71.9 | Lambs | CRD | FA | Tunnel |  |  |  |  |  | Intensive |  |
| Wood et al. (2009) | 95.9 | Lambs | CRD | Encapsulated FA | Tunnel |  |  |  |  |  | Intensive |  |
| Van Zijderveld et al. (2011) | 25.0 | Dairy cattle | RCBD | Calcium fumarate | RC | 66 | 34 | 3.3 | 16.7 | 41.5 | Intensive | Grass silage and corn silage |
| Yang et al. (2012) | 15.4 | Goat | LSD | Sodium fumarate | RC | 41 | 59 |  | 9.7 | 35.3 | Intensive | wildrye hay |
| Yang et al. (2012) | 15.4 | Goat | LSD | Sodium fumarate | RC | 58 | 42 |  | 9.7 | 44.4 | Intensive | wildrye hay |
| Li et al. (2018) | 22.2 | Goat | LSD | FA | RC | 49.3 | 50.7 |  | 14.2 | 38.3 | Intensive | Alfalfa hay, corn silage |
| Li et al. (2018) | 22.2 | Goat | LSD | FA | RC | 49.3 | 50.7 |  | 14.3 | 38.5 | Intensive | Alfalfa hay, corn silage |
| Li et al. (2021) | 20.0 | Goat | RCBD | FA | RC | 52.1 | 47.9 | 4.1 | 18.6 | 36.1 | Intensive | Alfalfa hay, corn silage |
| Maigaard et al. (2024) | 17.0 | Dairy cattle | LSD | FA | RC | 56 | 44 | 2.6 | 17.2 | 32.3 | Intensive | Grass clover and corn silage |
| Maigaard et al. (2024) | 21.3 | Dairy cattle | LSD | FA | RC | 56 | 44 | 3.3 | 16 | 31.6 | Intensive | Grass clover and corn silage |
| Newbold et al. (2002) | 20.0 | Lambs | LSD | FA | RC |  |  |  |  |  | Intensive | Grass hay |
| Newbold et al. (2002) | 40.0 | Lambs | LSD | FA | RC |  |  |  |  |  | Intensive | Grass hay |
| Newbold et al. (2002) | 80.0 | Lambs | LSD | FA | RC |  |  |  |  |  | Intensive | Grass hay |
| Dong et al. (2025) | 20.6 | Goat | RCBD | Fumarate | RC | 48 | 52 | 4.1 | 18.5 | 36.1 | Intensive | Corn Silage, alfalfa hay |

DOE, design of experiment; CRD, completely randomized design; FA, fumaric acid; LSD, Latin square design; RCBD, randomized control block design; RC, respiratory chamber; SF6, sulfur hexafluoride.

**Supplementary Table S2**

The descriptive characters of the studies included in meta-analysis.

| Character, % unless mentioned | Animal | N | Missing | Mean | SD | Minimum | Maximum |
| --- | --- | --- | --- | --- | --- | --- | --- |
| Forage | Beef cattle | 3 | 0 | 83.3 | 14.4 | 75.0 | 100.0 |
| Forage | Dairy cattle | 4 | 0 | 69.5 | 20.8 | 56.0 | 100.0 |
| Forage | Lambs | 4 | 5 | 95.0 | 0.00 | 95.0 | 95.0 |
| Forage | Goat | 5 | 0 | 49.6 | 6.13 | 41.0 | 58.0 |
| Concentrate | Beef cattle | 3 | 0 | 16.6 | 14.4 | 0.00 | 25.0 |
| Concentrate | Dairy cattle | 4 | 0 | 30.5 | 20.8 | 0.00 | 44.0 |
| Concentrate | Lambs | 4 | 5 | 5.00 | 0.00 | 5.00 | 5.00 |
| Concentrate | Goat | 5 | 0 | 50.3 | 6.13 | 42.0 | 59.0 |
| FA dose g/kg DMI | Beef cattle | 3 | 0 | 17.8 | 7.82 | 9.20 | 24.4 |
| FA dose g/kg DMI | Dairy cattle | 4 | 0 | 27.9 | 14.0 | 17.0 | 48.4 |
| FA dose g/kg DMI | Lambs | 9 | 0 | 65.3 | 27.3 | 20.0 | 100.0 |
| FA dose g/kg DMI | Goat | 5 | 0 | 19.0 | 3.44 | 15.40 | 22.2 |
| EE | Beef cattle | 1 | 2 | 2.10 | 0.00 | 2.10 | 2.10 |
| EE | Dairy cattle | 4 | 0 | 3.03 | 0.34 | 2.60 | 3.30 |
| EE | Lambs | 4 | 5 | 2.20 | 0.00 | 2.20 | 2.20 |
| EE | Goat | 1 | 4 | 4.10 | 0.00 | 4.10 | 4.10 |
| CP | Beef cattle | 3 | 0 | 11.9 | 4.48 | 6.80 | 14.6 |
| CP | Dairy cattle | 4 | 0 | 17.4 | 1.66 | 16.0 | 19.8 |
| CP | Lambs | 4 | 5 | 17.6 | 0.00 | 17.6 | 17.6 |
| CP | Goat | 5 | 0 | 14.1 | 3.74 | 9.70 | 18.6 |
| NDF | Beef cattle | 2 | 1 | 35.4 | 1.06 | 34.7 | 36.2 |
| NDF | Dairy cattle | 4 | 0 | 36.6 | 5.46 | 31.6 | 41.5 |
| NDF | Lambs | 4 | 5 | 40.8 | 0.00 | 40.8 | 40.8 |
| NDF | Goat | 5 | 0 | 38.1 | 3.57 | 35.3 | 44.4 |

EE, ether extract; CP, crude protein of the diet; FA, fumaric acid; NDF, neutral detergent fibre; N, number of effect sizes.


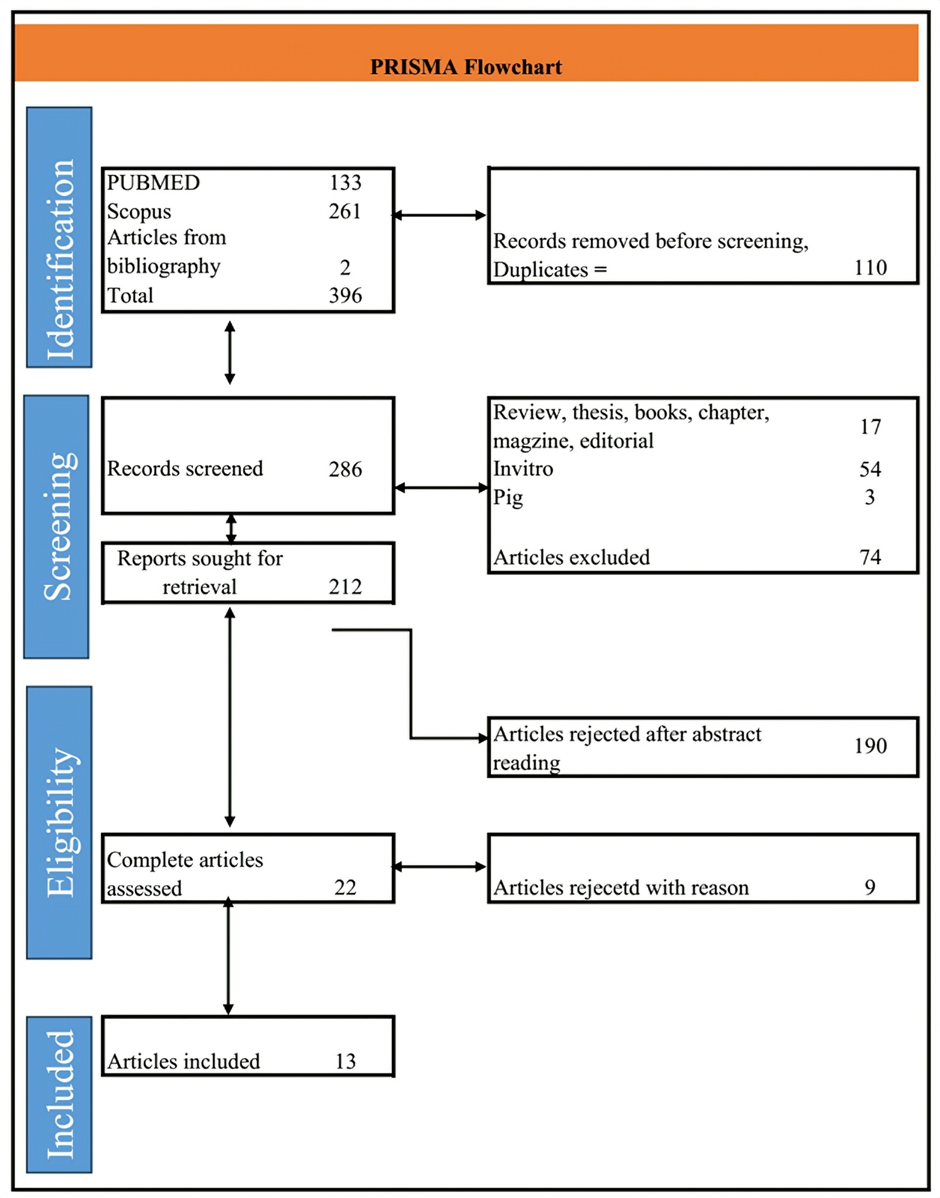


**Supplementary Figure S1.** The PRISMA flow diagram illustrates the selection process for studies included in this meta-analysis.


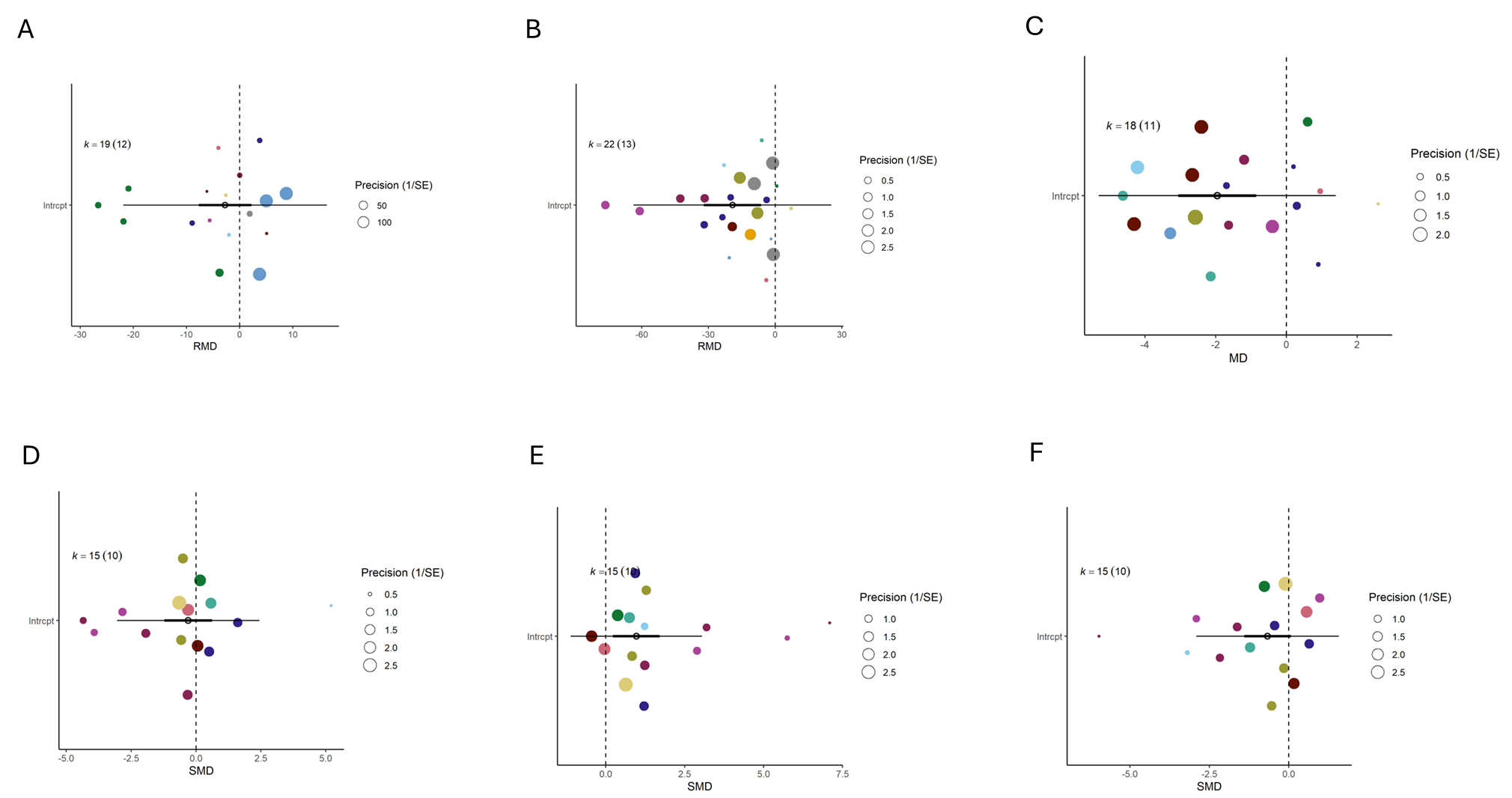


**Supplementary Figure S2.** The orchard plot for DMI (A), methane production (B), methane yield (C), acetate (D), propionate (F) and butyrate (F), the overall estimate from a multilevel random-effects meta-analysis, with a 95% confidence interval (CI) that spans the line of no effect (dotted line). The thick black horizontal line represents the prediction interval, while the dotted vertical line represents the line of no effect.


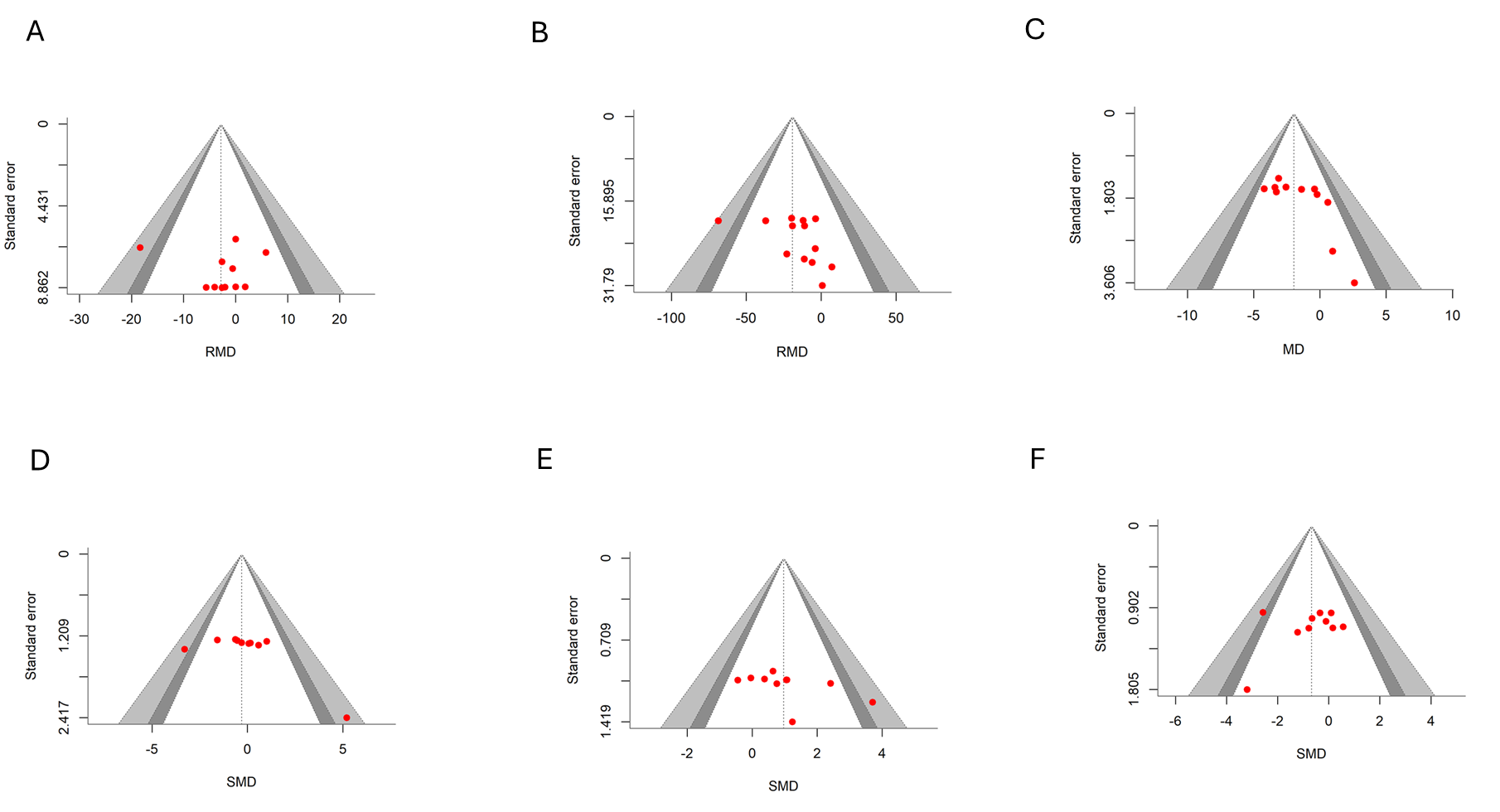


**Supplementary Figure S3.** The contour-enhanced funnel plot for studies included DMI (A), methane production (B), methane yield (C), acetate (D), propionate (E) and butyrate (F), the symmetrical distribution of effect sizes around the standard error, indicates no bias, while asymmetrical distribution indicates biasness in meta-analysis.

**References**

Bayaru, E., S. Kanda, T. Kamada, H. ITABASHI, S. ANDOH, T. NISHIDA, M. ISHIDA, T. ITOH, K. NAGARA, and Y. ISOBE. 2001. Effect of fumaric acid on methane production, rumen fermentation and digestibility of cattle fed roughage alone. Nihon Chikusan Gakkaiho 72(2):139-146.

Beauchemin, K., and S. McGinn. 2006. Methane emissions from beef cattle: Effects of fumaric acid, essential oil, and canola oil. J. Anim. Sci. 84(6):1489-1496.

Dong, H., X. Zhang, S. Li, J. Ma, J. Zhang, H. Chen, Y. lamothe Crespo, J. Yao, and Z. Li. 2025. Effect of fumarate and live yeast on ruminal fermentation, methane emissions, and blood metabolites in dairy goats. J. Dairy Sci. 108:10855–10868.

Kolver, E., and P. Aspin. 2006. Supplemental fumarate did not influence milk solids or methane production from dairy cows fed high quality pasture. In: Proceedings of the 66th Conference of the New Zealand Society of Animal Production,, Napier, New Zealand

Li, Z., X. Lei, X. Chen, Q. Yin, J. Shen, and J. Yao. 2021. Long-term and combined effects of N-[2-(nitrooxy) ethyl]-3-pyridinecarboxamide and fumaric acid on methane production, rumen fermentation, and lactation performance in dairy goats. Journal of Animal Science and Biotechnology 12(1):125.

Li, Z., N. Liu, Y. Cao, C. Jin, F. Li, C. Cai, and J. Yao. 2018. Effects of fumaric acid supplementation on methane production and rumen fermentation in goats fed diets varying in forage and concentrate particle size. Journal of animal science and biotechnology 9(1):21.

Maigaard, M., M. R. Weisbjerg, A. L. F. Hellwing, M. Larsen, F. B. Andersen, and P. Lund. 2024. The acute effects of rumen pulse-dosing of hydrogen acceptors during methane inhibition with nitrate or 3-nitrooxypropanol in dairy cows. J. Dairy Sci. 107(8):5681-5698.

McGinn, S., K. Beauchemin, T. Coates, and D. Colombatto. 2004. Methane emissions from beef cattle: Effects of monensin, sunflower oil, enzymes, yeast, and fumaric acid. J. Anim. Sci. 82(11):3346-3356.

Molano, G., T. Knight, and H. Clark. 2008. Fumaric acid supplements have no effect on methane emissions per unit of feed intake in wether lambs. Australian Journal of Experimental Agriculture 48(2):165-168.

Newbold, C., J. Ouda, S. López, N. Nelson, H. Omed, R. Wallace, and A. Moss. 2002. Propionate precursors as possible alternative electron acceptors to methane in ruminal fermentation. In: Greenhouse Gases and Animal Agriculture: Proceedings of the 1st International Conference on Greenhouse Gases and Animal Agriculture, Obihiro, Japan, 7-11 November, 2001. p 151-154.

Van Zijderveld, S., J. Dijkstra, H. Perdok, J. Newbold, and W. Gerrits. 2011. Dietary inclusion of diallyl disulfide, yucca powder, calcium fumarate, an extruded linseed product, or medium-chain fatty acids does not affect methane production in lactating dairy cows. J. Dairy Sci. 94(6):3094-3104.

Wood, T., R. Wallace, A. Rowe, J. Price, D. R. Yáñez-Ruiz, P. Murray, and C. Newbold. 2009. Encapsulated fumaric acid as a feed ingredient to decrease ruminal methane emissions. Anim. Feed Sci. Technol. 152(1-2):62-71.

Yang, C., S. Mao, L. Long, and W. Zhu. 2012. Effect of disodium fumarate on microbial abundance, ruminal fermentation and methane emission in goats under different forage: concentrate ratios. Animal 6(11):1788-1794.
